# Interferon regulatory factor 7 regulates airway epithelial cell responses to human rhinovirus infection

**DOI:** 10.1186/s12864-016-2405-z

**Published:** 2016-01-25

**Authors:** Anthony Bosco, Shahina Wiehler, David Proud

**Affiliations:** Telethon Kids Institute, University of Western Australia, PO Box 855, West Perth, WA 6872 Australia; Airway Inflammation Research Group, Snyder Institute for Chronic Diseases, and the Department of Physiology & Pharmacology, University of Calgary Faculty of Medicine, Calgary, AB Canada

**Keywords:** Rhinovirus, Airway epithelial cells, Interferon regulatory factor 7, Innate immunity, Gene expression profiling, Gene silencing

## Abstract

**Background:**

Human rhinoviruses (HRV) cause the majority of colds and trigger exacerbations of chronic lower airway diseases. Airway epithelial cells are the primary site for HRV infection and replication, and the initiation of host inflammatory responses. At present, the molecular mechanisms that underpin HRV responses in airway epithelial cells are incompletely understood. The aim of this study was to employ microarray profiling, upstream regulator analysis, and siRNA mediated gene silencing to further our understanding of the role of interferon regulatory factor 7 (IRF7) in this response.

**Methods:**

Primary human bronchial epithelial cells (HBE) where transfected with siRNA that targets IRF7 or a non-silencing control (all-star control) using Lipofectamine. The cells were allowed to recover, and then cultured in the presence or absence of HRV-16 for 24 h. Global patterns of gene expression were profiled on microarrays. A subset of genes identified in the microarray study were validated at the mRNA and/or protein level using real time RT-qPCR, ELISA, and western blots.

**Results:**

Hundreds of genes were upregulated in HBE during HRV infection. Pathways analysis demonstrated that these genes were mainly involved in type I and II interferon signaling, RIG-I/MDA5 signaling, antigen processing and presentation, and apoptosis. Upstream regulator analysis of these data suggested that IRF7 was a major molecular driver of this response. Knockdown of IRF7 reduced the HRV-driven upregulation of genes involved in antiviral responses (interferon signaling, Toll-like receptor signaling, NOD-like receptor signaling, RIG-I/MDA5 signaling), and increased the expression of genes that promote inflammation (e.g. CXCL5, IL-33, IL1RL1) and the response to oxidative stress. However, the majority of genes that were perturbed by HRV in HBE cells including those that are known to be regulated by IRF7 were insensitive to IRF7 knockdown. Upstream regulator analysis of the part of the response that was insensitive to IRF7 knockdown suggested it was driven by NF-κB, STAT1, STAT3, and IRF1.

**Conclusions:**

Our findings demonstrate that IRF7 regulates the expression of genes involved in antiviral immunity, inflammation, and the response to oxidative stress during HRV infections in HBE cells, and also suggests that other transcription factors play a major role in this response.

**Electronic supplementary material:**

The online version of this article (doi:10.1186/s12864-016-2405-z) contains supplementary material, which is available to authorized users.

## Background

Human rhinovirus is a positive sense, single-strand RNA virus from the Picornavirus family. It causes the majority of colds, and it also triggers exacerbations of asthma, chronic obstructive pulmonary disease, and cystic fibrosis [1]. More than 150 strains of HRV have been identified, and these have been classified into three species (A, B, C) based on genome sequencing data [2]. Airway epithelial cells are the primary site for rhinovirus infection and replication. Depending on the serotype of HRV, cell entry is mediated by binding to the receptors ICAM-1 (major group), low-density-lipoprotein receptor family members (minor group), or cadherin-related family member 3 (CDHR3 for HRV-C) [3].

Host responses to HRV are thought to be initiated when viral proteins and nucleic acids trigger pathogen recognition receptors (PRR) of the innate immune system (TLR2, TLR3, TLR7, TLR8, MDA5, RIG-I) [4–6]. PRR signaling triggers intracellular signaling cascades that converge on the IRF and NF-κB family of transcription factors, which in turn activate interferon-induced antiviral and proinflammatory gene expression programs respectively. In epithelial cells, interferon programs are mainly activated via TLR3, MDA5, and RIG-I signaling [4–6]. IRF7 plays a central role in the activation of innate antiviral responses by controlling the transcription of type I and III interferon genes [7]. Type I interferon signaling in turn upregulates IRF7 expression, thereby providing positive feedback amplification of the antiviral response [8]. The interferon system plays a crucial role in antiviral defence by upregulating a set of effector molecules (e.g. Mx1, OAS, PKR, ISG15), which induce a robust antiviral state in infected and neighbouring cells preventing the spread of infection [9]. The importance of IRF7 in antiviral immunity was clearly demonstrated in IRF7 deficient mice, which are highly susceptible to viral infections [10]. Recent data in humans indicates that null mutations in IRF7 are associated with susceptibility to severe, life-threatening influenza [11].

Although it is well established that IRF7 is a master regulator of the antiviral response, the function (or redundancy thereof) of IRF7 can vary markedly depending on the cell type and the virus [12]. We reported that expression of IRF7 was upregulated in nasal epithelial scrapings from human adults after experimental infection with HRV-16 [13]. Employing a computational analysis of gene expression profiling data derived from nasal wash samples, we also showed that IRF7 was a major hub, connecting interferon-related gene networks during picornavirus-induced asthma exacerbations in children [14]. Lewis et al. reported that expression of IRF7 was upregulated in nasal aspirates from asthmatic children during natural colds [15]. Notably, all of the above studies focused on expression levels of IRF7; this is the first study to perform gene silencing experiments in HBE to elucidate the role of IRF7 in the regulation of HRV responses.

## Methods

### Materials

The following reagents were purchased from the indicated suppliers: Ham’s F-12 medium, Eagle’s minimal essential medium, Hank’s balanced salt solution (HBSS), penicillin-streptomycin-amphotericin B, L-glutamine, TRIzol reagent, sodium pyruvate, nonessential amino acids, gentamicin, fetal bovine serum (FBS), dNTPs, oligo(dT), random hexamers, and Superscript III from Invitrogen Life Technologies (Burlington, Ontario, Canada). Bronchial epithelial cell growth medium (BEGM) was from BioWhittaker (Walkersville, MD). Gene expression kits for GAPDH, IL-6, CXCL5, and HERC5 were from Applied Biosystems (Foster City, CA); antibody pairs for CCL5, CXCL5, and CXCL10, as well as recombinant standards for each assay were from R&D Systems (Minneapolis, MN); *Taq*Man master mix and complete protease inhibitor cocktail tablets were from Roche Diagnostics (Laval, Quebec, Canada); western blot antibody for IRF7 was from BioLegend (San Diego, CA), while the viperin antibody was from Abcam (Toronto, ON). All other chemicals were purchased from Sigma-Aldrich (St. Louis, MO).

### Primary epithelial cell culture

Normal human lungs not used for transplantation were obtained from a tissue retrieval service (International Institute for the Advancement of Medicine, Edison, NJ). Primary HBE cells from a total of 9 donors (4 male; age range 13–63 years) were used for the current studies. All donors died from head trauma or stroke, and none of the donors had any inflammatory lung disease. Written informed consent was obtained from responsible next of kin for tissues to be used for either transplant or for research. Approval to use recovered organs for these studies was obtained from the Conjoint Health Research Ethics Board of the University of Calgary. Primary HBE cells were obtained by protease digestion of dissected airways as previously described [16]. HBE cells were grown on six-well culture plates in BEGM. Hydrocortisone was removed from the medium 24 h prior to stimulation and all stimulations were performed in this hydrocortisone-free medium.

### Virus

WI-38 cells were purchased from the American Type Culture Collection (Manassas, VA). HRV-16 viral stocks were propagated in WI-38 cells and purified by centrifugation through sucrose to remove ribosomes and soluble factors of WI-38 origin as previously described [17]. HBE cells were infected with 10^5.5^ 50 % tissue culture-infective dose (TCID50) U/ml, which equates (approximately) to a multiplicity of infection of 1.

### RNA extraction and real-time RT-qPCR

Total cellular RNA was extracted from cultured epithelial cells with TRIzol (1 ml per 10 cm^2^) as previously described [18]. Yield of RNA was quantified based upon absorbance at 260 nm. RNA was DNase treated with DNase I (Ambion, Austin, TX) and then requantified. One μg of RNA was reverse transcribed with both Oligo dT and random hexamers with Superscript III, followed by PCR amplification in the presence of specific forward and reverse primers and fluorescently labeled probes for each gene of interest. Gene expression for CXCL10, CCL5, Viperin, IL-6, CXCL5, and HERC5 were quantified using the Applied Biosystems Model 7900 Sequence Detector (Foster City, CA). Analysis of the housekeeping gene GAPDH was performed on each sample using a primer and probe kit obtained from Applied Biosystems. Fold induction of each gene was calculated using the formula 2^-ΔΔCT^ as previously described [19]. Primers and probes for CXCL10, CCL5 and viperin were as follows: CXCL10 forward primer 5′-GAAATTATTCCTGCAAGCCAATTT-3′; reverse primer 5′-TCACCCTTCTTTTTCATTGTAGCA-3′; probe 5′-FAM-TCCACGTGTTGAGATCA-MGB-3′, CCL5 forward primer 5′-TCTGCGCTCCTGCATCTG-3′; reverse primer 5′-AGTGGGCGGGCAATGTAG-3′; probe 5′-FAM-ATTCCTCGGACACCACACCCTGCTG-MGB-3′, and viperin forward primer 5′-CCTGCTTGGTGCCTGAATCT-3′; reverse primer 5′- GCGCATATATTCATCCAGAATAAGG-3′; probe 5′-FAM-ACCAGAAGATGAAAGACT-MGB-3′. Gene expression kits were purchased from Applied Biosystems for IL-6 (Hs00985639_m1), CXCL5 (Hs01099660_g1), and HERC5 (Hs00180943_m1).

### ELISAs

CXCL10, CCL5 and CXCL5 protein levels were assayed by enzyme-linked immunosorbent assay using matched antibody pairs (R&D Systems, Minneapolis, MN).

### siRNA knockdown of IRF7

Subconfluent HBE cells were transfected with either 10 nM of a siRNA targeted to IRF7 or to a nontargeting siRNA, all-star control (Qiagen, Toronto, ON) for 24 h at 37 °C using Lipofectamine RNAiMAX (Life Technologies) in BEGM without antibiotics. After transfection, medium was changed to BEGM without hydrocortisone and cells were allowed to recover for 24 h. Cells were then infected with HRV-16, and supernatants, whole-cell lysates and total RNA were collected 24 h post infection. The specific forward siRNA sequences used were as follows: IRF7 siRNA #1, 5′-CCCGAGCTGCACGTTCCTATA-3′; IRF7 siRNA #2, 5′-CTGGAAGCACTTCGCGCGCAA-3′.

### Western blots

After stimulation, cells were washed with HBSS and then scraped in lysis buffer (1 % Triton X-100, 1X mini complete, 1 mM PMSF, 2 mM sodium orthovanadate, 20 mM sodium pyrophosphate, and 50 mM sodium fluoride). Lysates were incubated on ice for 10 min, frozen at −80 °C, thawed, sonicated, and centrifuged at 10,000 × g for 5 min at 4 °C. Protein concentrations for the Triton X-100-soluble lysates were quantified using a DC Protein Assay (Bio-Rad, Montreal, Quebec, Canada). Equivalent amounts of protein were separated by SDS-PAGE, and proteins were then transferred to a nitrocellulose membrane. Membranes were blocked with 5 % skim milk for 1 h and probed with either 1 μg/mL of the IRF7 antibody or a 1:1000 dilution of the viperin antibody overnight at 4 °C. Membranes were washed and then incubated for 1 h with a 1:10,000 dilution of horseradish peroxidase-conjugated anti-mouse Ig antibody (Jackson ImmunoResearch Laboratories, Inc., West Grove, PA). Proteins were visualized with a Pierce ECL substrate reagent (Thermo Scientific, Rockford, IL). Equal loading was further determined probing with an antibody against GAPDH.

### Gene expression profiling

Total RNA samples were shipped on dry ice to Expression Analysis Inc. (Durham, NC). Purified RNA was quantified using a Nanodrop spectrophotometer and quality was determined using the Agilent (Santa Clara, CA) 2100 Lab-on-a-Chip System. The RNA samples were processed using the Enzo Single Round RNA Amplification and Biotin Labeling System (Enzo, New York, NY), and hybridized to Primeview microarrays (Affymetrix, Santa Clara CA). The raw microarray data is available at the Gene Expression Omnibus repository under accession GSE70190.

The microarray data was analyzed in R software for statistical computing (https://www.r-project.org/). The quality of the microarray data was assessed with ArrayQualityMetrics [20]. The data was preprocessed employing the RMA algorithm [21]. A custom chip description file (primeviewhsentrezg, version 19) was used to map probe sets to genes [22]. The data was filtered to differentiate between relevant gene expression signals and noise [23]. Differentially expressed genes were identified using Linear models for microarray data (LIMMA), with False Discovery Rate (FDR) control for multiple testing [24]. Pathways analysis was performed using InnateDb. Upstream regulators were identified using Ingenuity Systems software [25]. The IRF7 gene network was reconstructed using experimentally supported findings from the Ingenuity Systems Knowledgebase [14].

### Statistical analysis

For assessments of mRNA and protein expression for individual genes of interest, normally distributed data were analyzed using one-way analysis of variance (ANOVA) with student Newman-Keuls post hoc analysis. Analysis of paired comparisons were performed using t-tests. Data that were not normally distributed were analyzed using Kruskal-Wallis ANOVA followed by Wilcoxon matched-pairs signed-rank test. Statistical hypotheses were two-sided and values of *p* < 0.05 were considered significant.

## Results

### Rhinovirus-induced gene expression patterns in human bronchial epithelial (HBE) cells

**Fig. 1:**
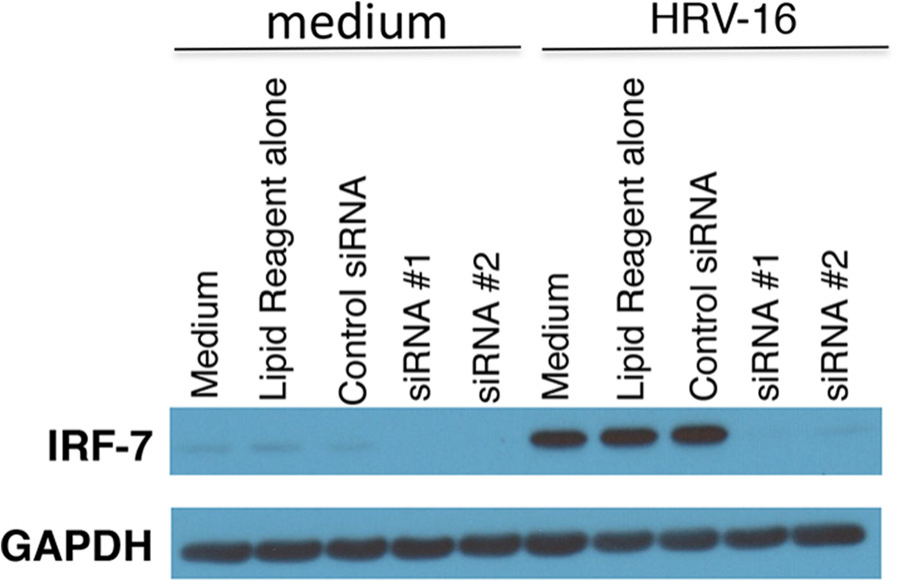
Knockdown of IRF7 protein in HBE cells. HBE cells were transfected with the transfection reagent only (lipid reagent alone), the all-star control siRNA (control siRNA), or two independent siRNAs that target IRF7 (siRNA #1, siRNA #2). The cells were allowed to recover, and then exposed to HRV. Protein expression was measured by Western Blot. Data are representative of *n* = 4

Primary HBE cells from 5 donors were transfected with siRNA that targets IRF7 or a non-silencing siRNA control (all-star control). The HBE cells were allowed to recover for 24 h, and then they were exposed to HRV or medium control for another 24 h. As illustrated in Fig. 1, HRV induced the expression of IRF7 protein in HBE cells that were transfected with control reagents (all-star control siRNA, lipid reagent alone, medium only), but not in HBE cells that were transfected with siRNA reagents that target IRF7 (siRNA #1, siRNA #2). Of note, IRF7 protein was not induced by the all-star control siRNA in HBE cells in the absence of HRV exposure.

**Fig. 2:**
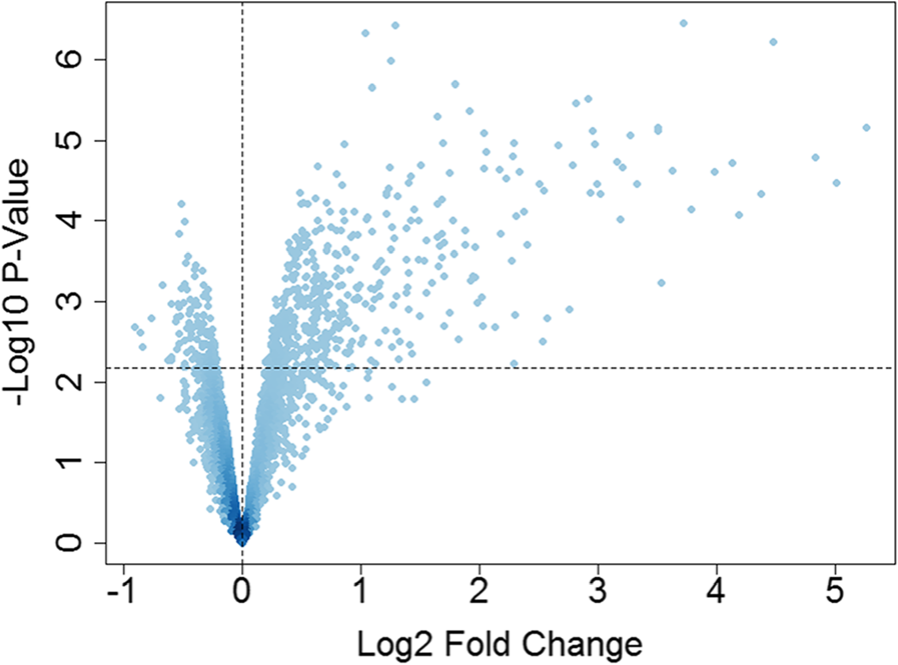
Gene expression patterns in HRV-induced HBE cells. HBE cells from 5 donors were transfected with a non-silencing siRNA (all-star control), allowed to recover, and then cultured in the presence or absence of HRV for 24 h. Gene expression was profiled on microarrays. The dashed horizontal line indicates FDR < 0.05

Next, global patterns of gene expression were profiled on microarrays. Initially, we analyzed the data pertaining to the response to HRV in HBE cells transfected with the all-star control. As illustrated in Fig. 2, 437 genes were upregulated in the response and 139 genes were downregulated (False Discovery Rate (FDR) < 0.05, Additional file 1). The biological function of the differentially expressed genes was interrogated using InnateDb. The data showed the upregulated genes were mainly involved in type I and II interferon signaling, RIG-I/MDA5 signaling, antigen processing and presentation, and apoptosis (Additional file 2). The downregulated genes were not strongly enriched for immune-related pathways, therefore we utilized the Gene Ontology (GO) database to interrogate their function. This analysis showed that the downregulated genes were enriched with the GO biological process term epithelial development (GO0060429; ALDH3A2, CTGF, DKK1, EMP1, ERRFI1, INSR, JAG1, KRT15, PTHLH, SCEL, SOX21; adjusted *p*-value = 0.0079).

### IRF7 is a putative molecular driver of interferon-induced gene networks

**Fig. 3:**
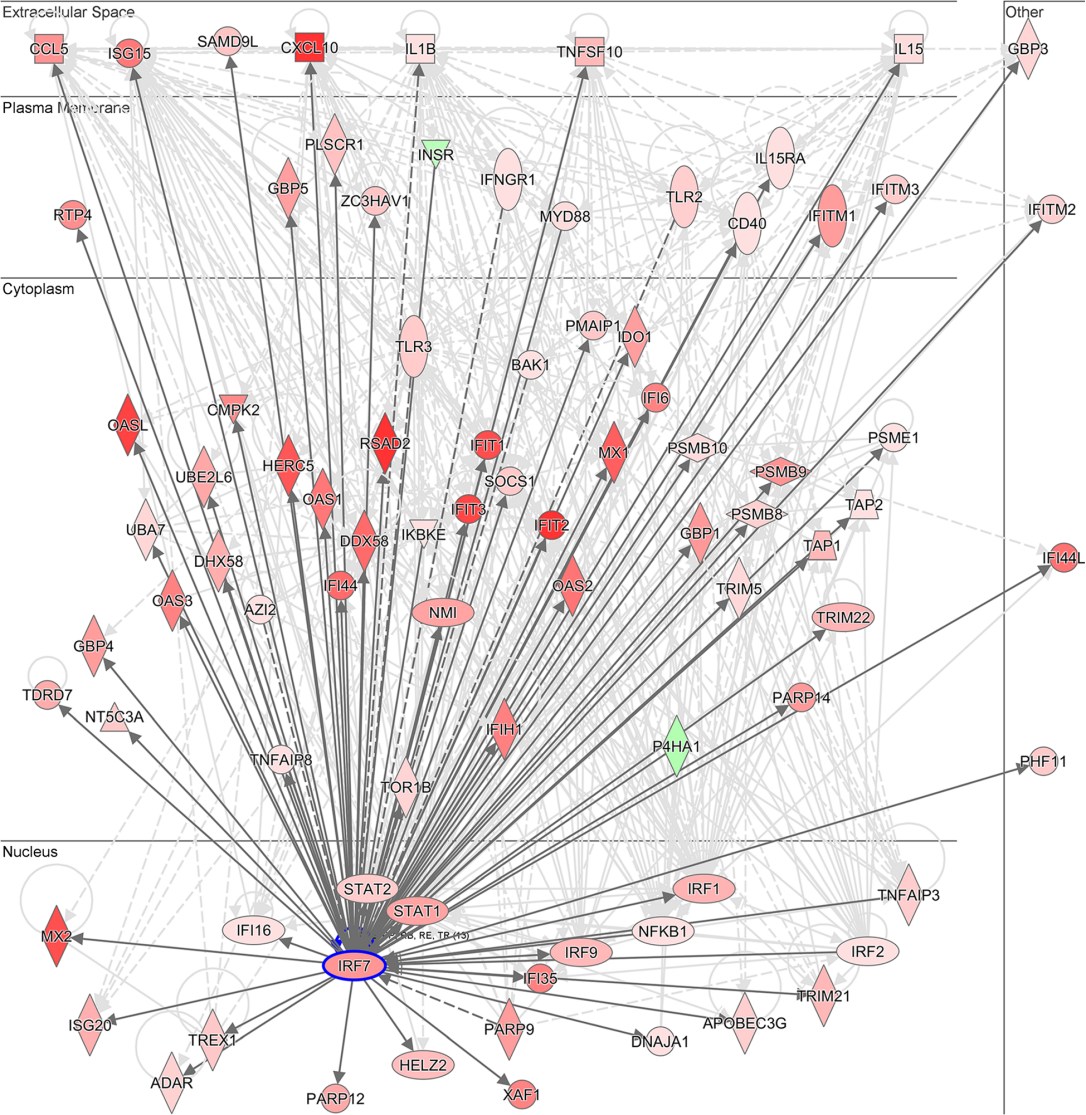
IRF7 is a putative molecular driver of HBE responses to HRV. Differentially expressed genes were identified in HRV-induced HBE cells (Fig. 2), and then the wiring diagram of the IRF7 gene network was reconstructed from these data employing experimentally supported findings from published studies. Solid and dashed lines indicate direct and indirect interactions respectively. Genes colored red were upregulated in the response and genes colored green were downregulated

Upstream regulator analysis was employed to infer the molecular drivers that give rise to HRV-induced gene expression patterns in HBE cells (transfected with the all-star control) [25]. In this analysis, the differentially expressed genes from Fig. 2 were tested for enrichment of downstream target genes of known transcriptional regulators and other signal transduction pathways. As illustrated in Table 1, the most significant candidate drivers were IFNG, IFNA2, IRF7, TNF, and IFNL1. Notably, IRF7 was the only driver amongst these candidates that was also upregulated in the response (Additional file 1). To illustrate the role of IRF7 in the regulation of HRV responses, we utilized experimentally supported findings from the Ingenuity Systems database to reconstruct the IRF7 network [14]. The resulting network is shown in Fig. 3.

**Table 1:** Identification of molecular drivers of gene expression patterns in HRV-induced HBE cells

Upstream regulator	Predicted activation state	Activation Z-score	*P*-value of overlap
IFNG	Activated	10.890	1.08E-90
IFNA2	Activated	8.106	9.79E-85
IRF7	Activated	8.284	1.76E-71
TNF	Activated	9.828	3.92E-67
IFNL1	Activated	6.936	1.02E-62
IFNAR	Activated	6.306	5.99E-55
STAT1	Activated	6.317	2.32E-54
IFNB1	Activated	5.436	3.64E-50
STAT3	Activated	4.185	9.25E-48
IRF1	Activated	5.780	6.24E-46
NFkB (complex)	Activated	6.850	1.41E-39
TLR3	Activated	5.327	5.45E-39
IL1B	Activated	6.926	1.29E-37
CD40LG	Activated	4.739	8.56E-35
OSM	Activated	5.463	1.99E-34
TLR4	Activated	5.165	3.64E-33
NKX2-3	Inhibited	−7.703	1.50E-58
MAPK1	Inhibited	−6.867	5.29E-58
TRIM24	Inhibited	−6.002	1.67E-44
IL1RN	Inhibited	−6.336	2.84E-35

### Knockdown of IRF7 perturbs HBE responses to rhinovirus

We next examined the impact of IRF7 knockdown on the HRV response. Gene expression patterns were compared between HRV-induced HBE cells transfected with IRF7-siRNA #1 versus the all-star control. The data showed that knockdown of IRF7 resulted in decreased expression of 447 genes, and increased expression of 181 genes (Additional file 3, Fig. 4). The downregulated genes were mainly involved in type I and II interferon signaling, Toll-like receptor signaling, NOD-like receptor signaling, and RIG-I/MDA5 signaling (Additional file 4). As a supplementary analysis, we also examined the function of the downregulated genes after removal of known IRF7 target genes. These residual genes were associated with NOD-like receptor signaling pathways, trafficking of connexons, and metabolic pathways (Additional file 5). The upregulated genes were involved in the response to oxidative stress (GO:0006979; ADA, ARL6IP5, CYGB, FOS, MMP14, NET1, NQO1, PLK3, SNCA, TRPC6, UCP2; *p*-value = 0.0004) and inflammation (GO:0050729; ADORA2B, C3, IL1RL1, IL33, SERPINE1, TNFSF11; *p*-value = 0.0005), although it is noteworthy that these findings were not significant after adjustment of the *p*-values for multiple testing.

**Fig. 4:**
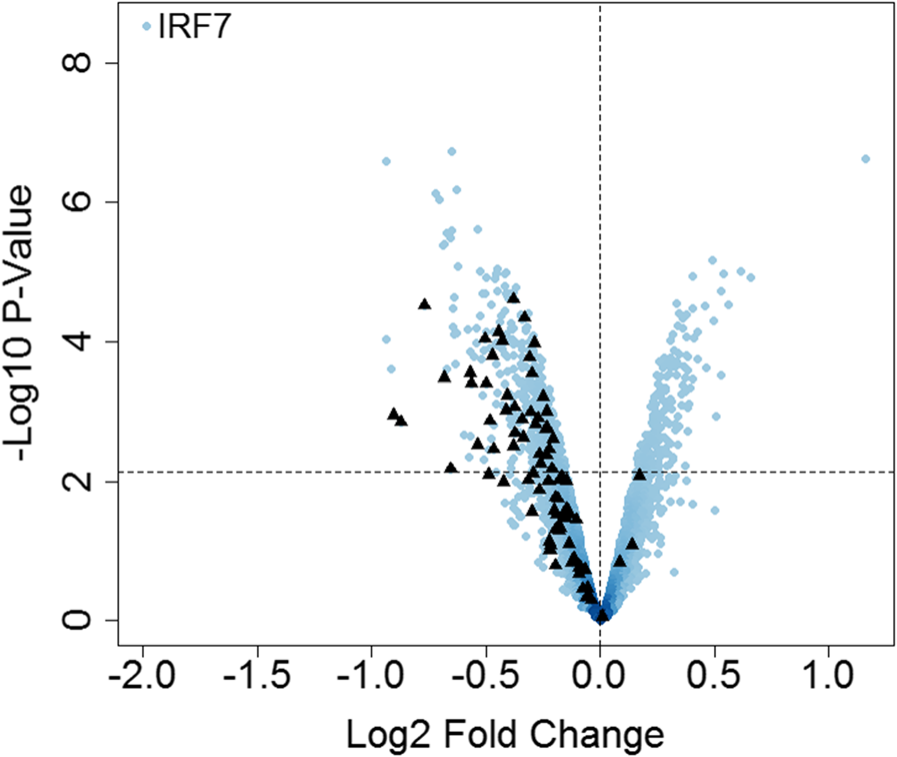
Knockdown of IRF7 perturbs HBE cells responses to HRV. HBE cells from five donors were transfected with siRNA that targets IRF7 (siRNA #1) or the non-silencing all-star control, allowed to recover, and then exposed to HRV for 24 h. Gene expression was profiled on microarrays. The data was compared between HRV-induced cells treated with IRF7-siRNA #1 versus the all-star control. Genes with negative values on the horizontal axis were decreased by IRF7 knockdown, whereas those with positive values were increased. The black triangles show the location of genes from the IRF7 network. The dashed horizontal line indicates FDR < 0.05

**Fig. 5:**
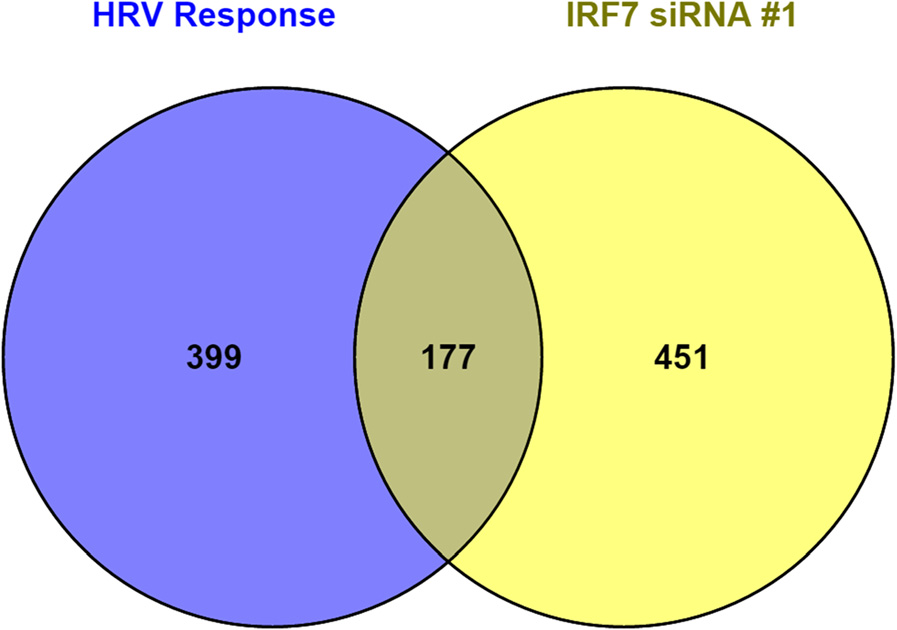
Most HRV responsive genes were insensitive to IRF7 knockdown. Venn diagram illustrating the overlap between those genes that were modulated by HRV (from Fig. 2) versus those that were sensitive to IRF7 knockdown (from Fig. 4)

We next wanted to investigate the part of the HRV response that was insensitive to IRF7 knockdown. In HBE cells that were transfected with the all-star control siRNA, 576 genes were differentially expressed after HRV exposure, but only 177 of these genes (31 %) were sensitive to IRF7 knockdown (Fig. 5). Moreover, the IRF7 gene network depicted in Fig. 3 contained 86 genes, and only 41 of these (47 %) were sensitive to IRF7 knockdown (Fig. 4, Additional file 6). We analyzed these HRV responsive genes that were insensitive to IRF7 knockdown using upstream regulator analysis. The data showed the highest ranking candidate transcription factors driving this response were NF-κB, STAT1, STAT3, and IRF1 (Additional file 7).

### Validation of the microarray findings

**Fig. 6:**
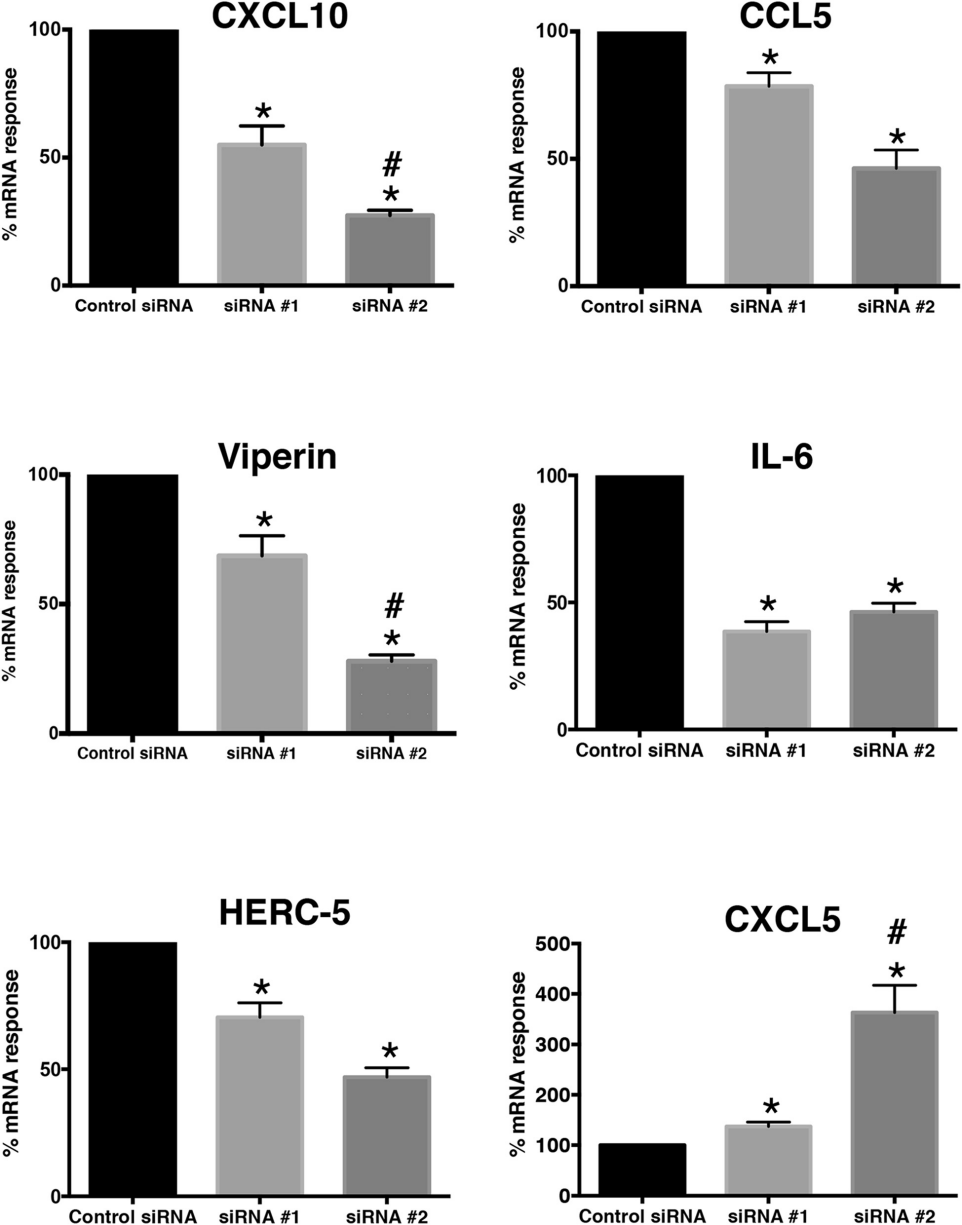
Validation of the microarray data at the mRNA level. HBE cells were transfected with the all-star control siRNA, or two independent siRNA reagents that target IRF7 (siRNA #1, siRNA #2). The cells were allowed to recover, and then exposed to HRV for 24 h. Gene expression levels were measured by real time RT-qPCR. The data for siRNA #1 and siRNA #2 are expressed as percent mRNA response relative to the all-star control. Data are mean ± SEM from 4 experiments. *Asterisks* show significant differences compared to the all star control siRNA treatment. *Hashmarks* (#) indicate significant differences between siRNA#1 and siRNA#2

**Fig. 7:**
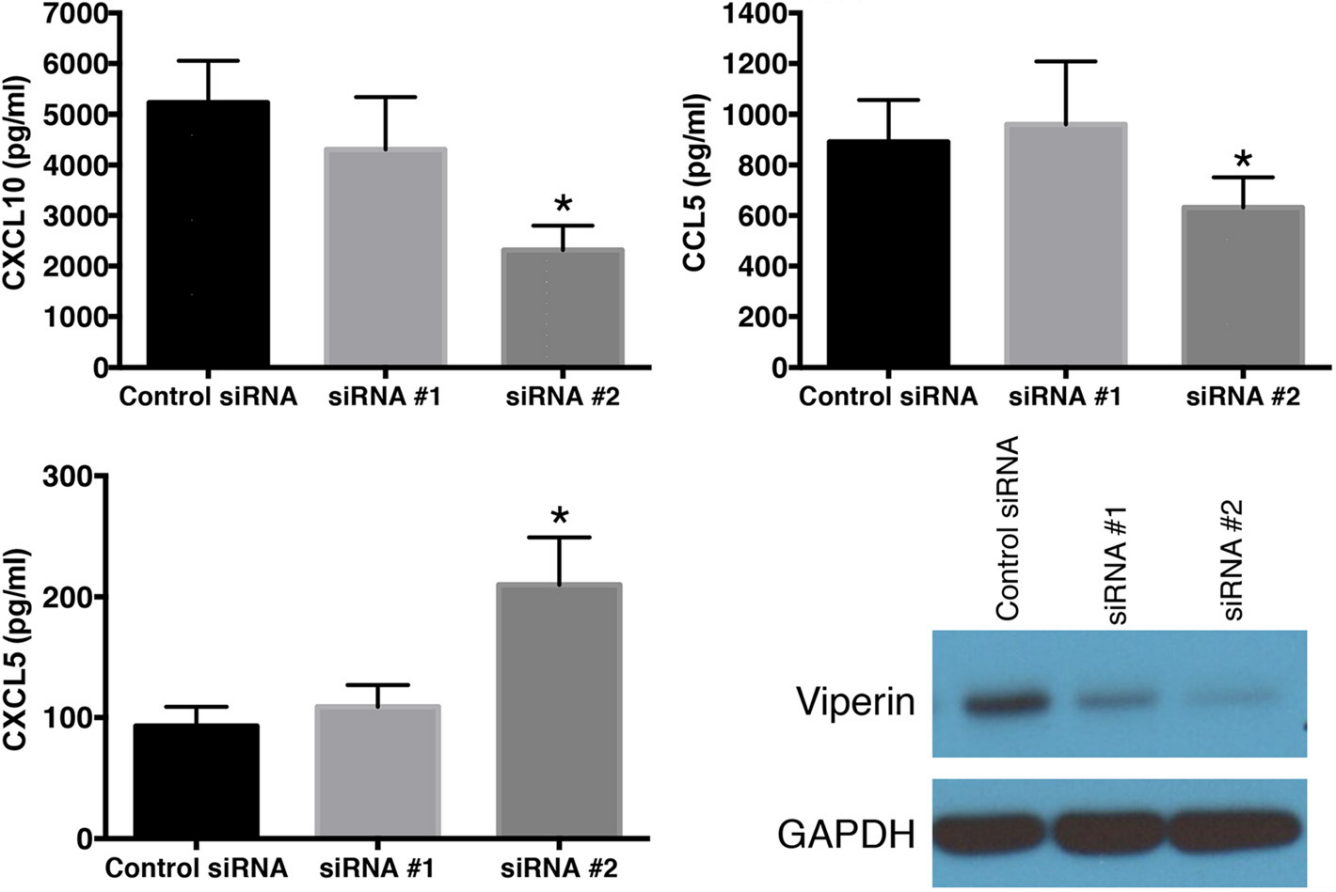
Validation of the microarray data at the protein level. HBE cells were transfected with the all-star control siRNA, or two independent siRNA reagents that target IRF7 (siRNA #1, siRNA #2). The cells were allowed to recover, and then exposed to HRV for 24 h. Protein levels for CCL5, CXCL5, CXCL10 were measured by ELISA and are shown as mean ± SEM from 4 experiments. *Asterisks* show significant differences compared to the all star control siRNA treatment. Expression of viperin protein was assessed by western blot. Data are representative of 4 such experiments

To confirm the findings from the microarray experiment, a subset of genes were selected for validation at the mRNA and/or protein levels. As illustrated in Fig. 6, Reverse Transcription quantitative PCR (RT-qPCR) analysis demonstrated that knockdown of IRF7 in HBE cells followed by exposure to HRV resulted in reduced expression of CCL5, CXCL10, HERC5, IL-6, RSAD2 (viperin), and increased expression of CXCL5, and this was consistent with the microarray data (Additional file 3). Expression levels of CCL5, CXCL5, CXCL10, and viperin were also examined at the protein level (Fig. 7). These data confirmed that the mRNA expression changes observed upon siRNA knockdown were associated with parallel changes at the protein level, although these changes were more pronounced with siRNA #2 than with siRNA #1.

## Discussion

IRF7 is a master regulator of type I interferon gene expression and antiviral immunity, but the role of IRF7 in HBE responses to HRV has not been previously investigated. Employing microarray profiling we showed that hundreds of genes were upregulated in HBE upon HRV infection. Upstream regulator analysis [25] suggested that IRF7 was a major molecular driver of this response. We then showed that knockdown of IRF7 in HBE altered HRV responses, by decreasing the upregulation of genes involved in type I and II interferon signaling and innate immunity (toll-like receptor signaling, NOD-like receptor signaling, RIG-I/MDA5 signaling), and increasing the expression of genes that promote inflammation (C3, CXCL5, IL-33, IL1RL1) and the response to oxidative stress. We validated a subset of the differentially regulated genes (CCL5, CXCL5, CXCL10, HERC5, IL-6, RSAD2/viperin) at the mRNA and/or protein levels, thus confirming the findings from the microarray experiment. The data also showed that only around 30 % of the HRV responsive genes and half of known IRF7 target genes were sensitive to IRF7 knockdown, and these genes exhibited modest fold changes in our experiments (less than 2 fold), suggesting that other transcription factors are likely to be important in the regulation of interferon gene expression programs in HBE cells. Upstream regulator analysis of the part of the HRV response that was insensitive to IRF7 knockdown suggested roles for NF-κB, STAT1, STAT3, and IRF1 in the regulation of the response. Overall, our findings demonstrate that IRF7 regulates the expression of genes involved in antiviral immunity, inflammation, and the response to oxidative stress in HBE cells during HRV infections, and also suggest that additional transcription factors play a major role in the regulation of this response.

We showed that the upregulation of CCL5, CXCL10, HERC5, IL-6, and RSAD2 by HRV was reduced by IRF7 knockdown in HBE cells. Regulation of these genes by IRF7 has been previously reported in other contexts [26–29]. CCL5 (RANTES) is a potent chemoattractant for T cells, monocytes, and eosinophils. CCL5 was upregulated in nasal wash samples from wheezing, HRV-infected infants in comparison to their nonwheezing counterparts [30]. CXCL10 is chemotactic for T cells and natural killer cells, and was strongly upregulated in airway epithelial cells during HRV infections [31]. CXCL10 promotes airways inflammation and airways hyperresponsiveness in mouse models of asthma [32]. HERC5 is an E3 protein ligase that mediates ISGylation of protein targets. It catalyzes the conjugation of ISG15 onto IRF3, which inhibits the ubiquitination and degradation of IRF3 by preventing IRF3 from interacting with Pin1. This results in the sustained activation of IRF3 and the antiviral response [33]. IL-6 promotes the acute phase response, stimulates T cell responses and antibody production, and it is increased during symptomatic rhinovirus infections [34]. IRF7 was required for IL-6 production by poly (I-C) stimulated THP-1 monocytes [29]. In contrast, IRF7 was dispensable for IL-6 production in plasmacytoid and myeloid dendritic cells stimulated by TLR7 or TLR9 ligands [10]. Viperin (RSAD2) is an antiviral protein that can inhibit influenza budding from the plasma membrane by interfering with lipid rafts [35]. Knockdown of viperin in airway epithelial cells increased HRV replication [13]. It is not possible to determine from our experiments if IRF7 regulates HRV-induced airway inflammatory responses in vivo, however, Girkin et al. showed that intranasal delivery of siRNA that targets IRF7 reduced the infiltration of neutrophils and macrophages in the bronchoalveolar lavage fluid of non-allergic mice after HRV-1B infection [36]. Taken together, these studies suggest that IRF7 regulates HRV-induced gene network patterns and ensuing cellular immune responses in the airways.

In contrast to genes involved in antiviral defense, knockdown of IRF7 resulted in increased expression of CXCL5, IL-33, and IL1RL1. CXCL5 (ENA-78) is important for neutrophil homeostasis and recruitment to the lung during infections [37]. IL-33 is a nuclear-associated alarmin expressed in the airway epithelium. It signals through the ST2 receptor (IL1RL1), which is highly expressed on T-helper type 2 (Th2) lymphocytes and group 2 innate lymphoid cells (ILC2s) [38]. IL-33 is upregulated in the airways during HRV-induced asthma exacerbations [39]. Moreover, supernatants from HRV-infected epithelial cells stimulate the production of Th2 cytokines by Th2 and ILC2 cells in an IL-33 dependant manner [39]. IRF7 binds to the IL-33 promoter and regulates IL-33 transcription in human monocyte THP-1 cell lines [40]. Given that IL-33 is a potent driver of asthmatic ILC2 and Th2 responses, and we found that knockdown of IRF7 results in upregulation of IL-33, this suggests the hypothesis that promoting IRF7 responses during asthma exacerbations may have a dual beneficial effect of increasing antiviral immunity and supressing IL-33 responses.

Knockdown of IRF7 decreased expression of genes involved in Nod-like receptor signaling, and increased expression of genes involved in the response to oxidative stress (e.g. cytoglobin (CYGB), NAD(P)H:quinone acceptor oxidoreductase 1 (NQO1), alpha-synuclein (SNCA), short transient receptor potential channel 6 (TRPC6)) [41–44]. In this context it is noteworthy that the generation of reactive oxygen species during HRV infection compromises epithelial barrier function, and this can lead to secondary bacterial infections [45, 46]. HRV infection stimulates the epithelial production of reactive oxygen species by triggering the dsRNA receptor Nod-like receptor X-1 (NLRX-1) [47]. Cross-talk between oxidative stress pathways and IRF7 signaling was reported in the context of respiratory syncytial virus infection [48]. The role of IRF7 in the regulation of Nod-like receptor signaling and oxidative stress pathways during HRV infections merits further investigation in follow-up studies.

Upstream regulator analysis demonstrated that IRF7 was the most significant candidate molecular driver that was upregulated in the HRV response. However, our siRNA studies showed that only about 30 % of the HRV responsive genes and half of known IRF7 target genes were sensitive to IRF7 knockdown. Whilst variable and incomplete knockdown and the potential for non-specific activation of antiviral pathways is a well-known limitation of siRNA technology, an alternative explanation of the data is that other transcription factors may be playing a role. Employing upstream regulator analysis we showed that NF-κB, STAT1, STAT3, and IRF1 were candidate drivers of the part of the HRV response that was insensitive to IRF7 knockdown. In this context it is noteworthy we have reported that HRV-induced upregulation of CXCL10 in bronchial epithelial cells was dependent on IRF1 [49]. Wang et al. have also reported that knockdown of IRF3 abrogated HRV-induced type I and III interferon responses in BEAS-2B epithelial cells [5]. Taken together, these observations suggest that interferon responses in HRV-induced HBE cells can be variably mediated by interactions between IRF1, IRF3, and IRF7.

This study has limitations that should be acknowledged. First, the gene expression studies were conducted at a single time point post infection (24 h), so it was not possible to determine the role of IRF7 in the kinetics of the response. Second, siRNA-mediated gene silencing was used to investigate the role of IRF7 in the regulation of gene expression patterns, and from this data it was not possible to determine if IRF7 was playing a direct or indirect regulatory role. Third, prior knowledge was used to infer the molecular drivers of the response, and this analysis cannot reveal novel molecular drivers. This latter limitation can be addressed using unbiased data-driven algorithms to infer the molecular drivers of the response [50]. Although we acknowledge that the use of epithelial cells grown in submersion culture, as opposed to differentiated air-liquid interface cultures, may be considered a limitation of the current study, we believe that the data obtained are still highly relevant. This is based on the observation that the profile of HRV-upregulated genes in these cultures, in both the present and in an earlier study [51], aligns closely with those observed in nasal epithelial scrapings from individuals experimentally infected with HRV in vivo [13].

## Conclusion

Our study demonstrates that IRF7 regulates the expression of antiviral, inflammatory and oxidative stress response pathways during HRV infections in HBE cells, and suggests that additional transcription factors play an important role in the response.

### Availability of supporting data

The microarray data are available at the Gene Expression Omnibus repository (accession GSE70190).

## Additional files

Additional file 1:
**This excel file contains the list of differentially expressed genes that were induced by HRV in HBE cells transfected with the all-star control siRNA.** (XLSX 71 kb) Additional file 2:
**This excel file contains the list of biological pathways that were upregulated by HRV in HBE cells transfected with the all-star control siRNA.** (XLSX 23 kb) Additional file 3:
**This excel file contains the list of differentially expressed genes identified in HRV-induced HBE cells transfected with IRF7-siRNA #1 versus the all-star control siRNA.** (XLSX 77 kb) Additional file 4:
**This excel file contains the list of biological pathways that were decreased in HRV-induced HBE cells transfected with IRF7-siRNA#1 versus the all-star control siRNA.** (XLSX 13 kb) Additional file 5:
**This excel file contains the results of a pathways analysis of remaining genes (after removal of known IRF7 target genes) that were decreased in HRV-induced HBE cells transfected with IRF7-siRNA#1 versus the all-star control siRNA.** (XLSX 11 kb) Additional file 6:
**This excel file contains the results of a differential gene expression analysis of IRF7 gene networks in HBE cells transfected with IRF7-siRNA#1 versus the all-star control siRNA.** (XLSX 18 kb) Additional file 7:
**This excel file contains the results from an Upstream regulator analysis of the HRV responsive genes in HBE cells that were insensitive to IRF7 knockdown.** (XLSX 11 kb)

## Abbreviations

ADA: adenosine deaminase
ADORA2B: adenosine A2b receptor
ALDH3A2: aldehyde dehydrogenase 3 family member A2
ANOVA: analysis of variance
ARL6IP5: ADP-ribosylation factor-like 6 interacting protein 5
BEGM: bronchial epithelial cell growth medium
C3: complement component 3
CCL5: chemokine (C-C motif) ligand 5, also known as regulated on activation, normal T cell expressed and secreted (RANTES)
CDHR3: Cadherin-related family member 3
CTGF: connective tissue growth factor
CXCL10: chemokine (C-X-C motif) ligand 10
CXCL5: chemokine (C-X-C motif) ligand 5
CYGB: cytoglobin
DKK1: dickkopf WNT signaling pathway inhibitor 1
ELISA: enzyme-linked immunosorbent assay
EMP1: epithelial membrane protein 1
ERRFI1: ERBB receptor feedback inhibitor 1
FBS: fetal bovine serum
FDR: False Discovery Rate
FOS: FBJ murine osteosarcoma viral oncogene homolog
GAPDH: glyceraldehyde-3-phosphate dehydrogenase
GO: Gene Ontology
HBE: human bronchial epithelial cells
HBSS: Hank’s balanced salt solution
HERC5: HECT and RLD domain containing E3 ubiquitin protein ligase 5
HRV: human rhinovirus
HRV-16: human rhinovirus type 16
HRV-C: human rhinovirus type C
IFNG: interferon gamma
IFNL1: interferon, lambda 1
IL-1RL1: interleukin 1 receptor-like 1
IL-33: interleukin-33
ILC2: group 2 innate lymphoid cells
INSR: insulin receptor
IRF7: interferon regulatory factor 7
ISG15: interferon stimulated gene that encodes interferon-induced 17 kDa protein
JAG1: jagged 1, KRT15, keratin 15 type I
LIMMA: linear models for microarray data
MDA5: melanoma differentiation associated gene 5
MMP14: matrix metallopeptidase 14
Mx1: MX dynamin-like GTPase 1
NET1: neuroepithelial cell transforming 1
NF-κB: nuclear factor kappa-light-chain-enhancer of activated B cells
NOD-like: receptor nucleotide-binding oligomerization domain receptor
NQO1: NAD(P)H dehydrogenase, quinone 1
OAS: 2′-5′-oligoadenylate synthetase 1
PKR: protein kinase RNA-activated
PLK3: polo-like kinase 3
PRR: pathogen recognition receptor
PTHLH: parathyroid hormone-like hormone
RANTES: regulated on activation, normal T cell expressed and secreted
RIG-I: retinoic acid-inducible gene 1
RT-qPCR: reverse transcription quantitative polymerase chain reaction
SCEL: sciellin
SDS-PAGE: sodium dodecyl sulfate polyacrylamide gel electrophoresis
SERPINE1: serpin peptidase inhibitor clade E
siRNA: small interfering RNA
SNCA: synuclein, alpha
SOX21: sex determining region Y box 21
Th2: T-helper type 2
TLR: toll-like receptor
TNF: tumor necrosis factor
TNFSF11: tumor necrosis factor (ligand) superfamily member 11, also known as receptor activator of nuclear factor-κB ligand (RANKL)
TRPC6: transient receptor potential cation channel, subfamily C, member 6
UCP2: uncoupling protein 2
